# Invasive *Andropogon gayanus* (Gamba grass) alters litter decomposition and nitrogen fluxes in an Australian tropical savanna

**DOI:** 10.1038/s41598-017-08893-z

**Published:** 2017-09-15

**Authors:** N. A. Rossiter-Rachor, S. A. Setterfield, L. B. Hutley, D. McMaster, S. Schmidt, M. M. Douglas

**Affiliations:** 1 Charles Darwin University, Darwin, Northern Territory 0909 Australia; 2 University of Western Australia, Perth, Western Australia 6099 Australia; 3 The University of Queensland, Brisbane, Queensland 4072 Australia

## Abstract

The African grass *Andropogon gayanus* Kunth. is invading Australian savannas, altering their ecological and biogeochemical function. To assess impacts on nitrogen (N) cycling, we quantified litter decomposition and N dynamics of grass litter in native grass and *A*. *gayanus* invaded savanna using destructive *in situ* grass litter harvests and litterbag incubations (soil surface and aerial position). Only 30% of the *A*. *gayanus in situ* litter decomposed, compared to 61% of the native grass litter, due to the former being largely comprised of highly resistant *A*. *gayanus* stem. In contrast to the stem, *A*. *gayanus* leaf decomposition was approximately 3- and 2*-*times higher than the dominant native grass, *Alloteropsis semilata* at the surface and aerial position, respectively. Lower initial lignin concentrations, and higher consumption by termites, accounted for the greater surface decomposition rate of *A*. *gayanus*. N flux estimates suggest the N release of *A*. *gayanus* litter is insufficient to compensate for increased N uptake and N loss via fire in invaded plots. Annually burnt invaded savanna may lose up to 8.2% of the upper soil N pool over a decade. Without additional inputs via biological N fixation, *A*. *gayanus* invasion is likely to diminish the N capital of Australia’s frequently burnt savannas.

## Introduction

Tropical savannas are globally significant, pyrogenic ecosystems^1^. Despite generally oligotrophic soils, savannas contribute 30% of the global terrestrial net productivity^2^. Nutrient release via litter decomposition is one of the key biogeochemical processes regulating plant productivity and nutrient cycling in tropical savannas^3,4^. The rate of decomposition is determined by abiotic factors such as temperature and moisture^5^ and biotic factors such as litter quality^3–6^. Climate seasonality is a major determinant of decomposition rates in wet-dry tropical savannas, with the highest rate of decomposition typically occurring during the wet season when moist and warm conditions suit decomposer communities^3,4,7–9^. Frequent fires impact on litter decomposition in savannas, with the quantity of litter available for decomposition in the wet season determined by the fire activity in the preceding dry season^7,10,11^. Fire intensity in particular determines the proportion of litter that volatilises during fires^12,13^.

Tropical savannas are impacted by invasive African C_4_ grasses^2,14,15^ including invasions in the llanos of Colombia and Venezuela^16^, the cerrado of Brazil^17^ and savanna woodlands of northern Australia^2,18^. It is critical to understand how these invasions, and the associated increase in grass biomass, could alter the critical pathway of N return to the soil, via litter decomposition, as this potentially has long-term implications for soil N pools. Past studies have found that in other ecosystems, high biomass invasive grasses tend to have higher rates of litter decomposition than native species, resulting in accelerated N cycling in invaded ecosystems^19,20^. This is generally attributed to differences in litter quantity and quality^19^, but may also be due to an altered decomposition microenvironment^19,21^.

**Figure 1:**
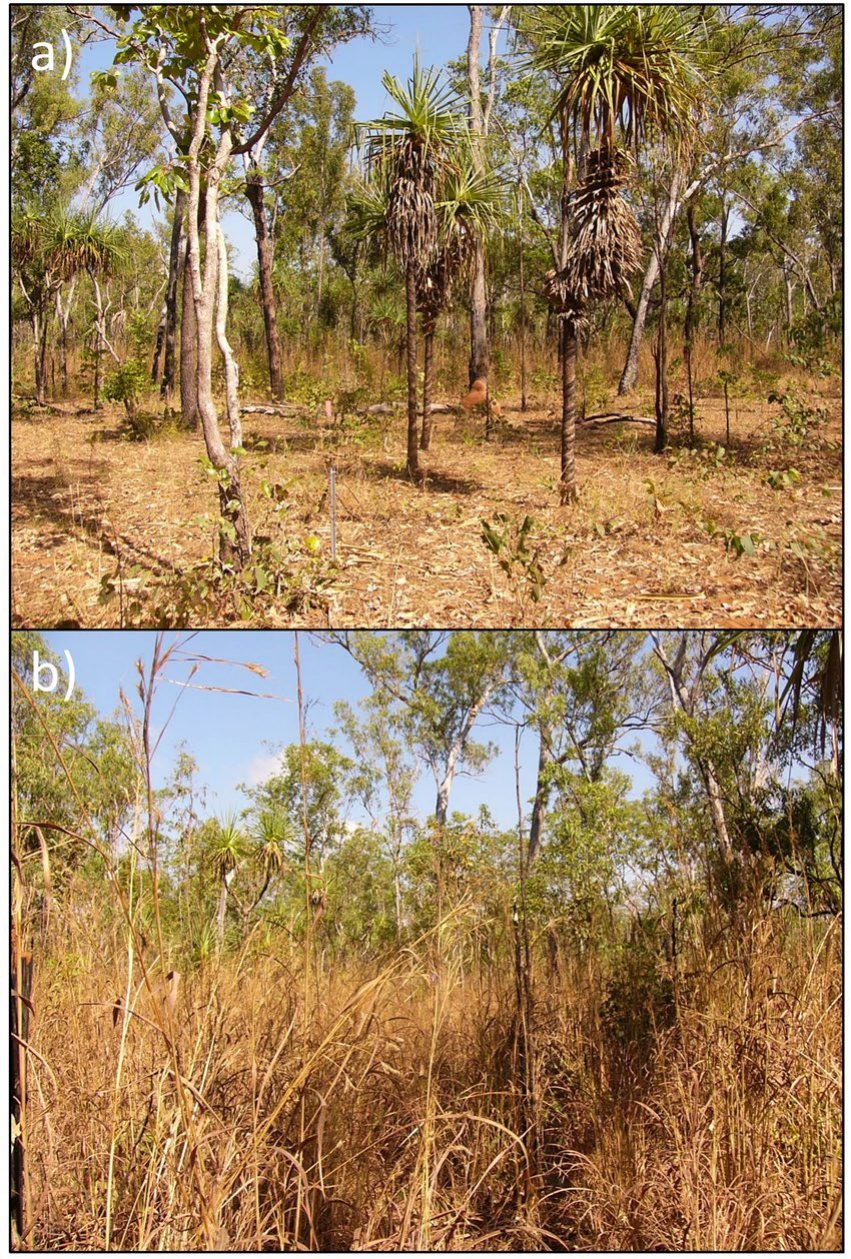
Changes to savanna structure with *A*. *gayanus* invasion. Photos of (**a**) Native grass savanna (dominated by *Alloteropsis semialata* and *Eriachne triseta*) and (**b**) *A*. *gayanus* invaded savanna; at Mary River National Park, in the late dry season (August). Photos were taken approximately 50 metres apart.

In northern Australia ∼10,000–15,000 km^2^ of savanna woodlands have been invaded by *A*. *gayanus* Kunth (gamba grass)^22^, converting diverse savanna grass understory^23^ into monospecific, tall swards of invasive grass^11^. *A*. *gayanus* alters the vegetation composition and structure^24,25^ and fire regimes^26,27^. We have previously documented that components of the N cycle are altered following invasion^28^, largely driven by the increases in biomass production and accompanying N pool in the wet season^11,13^. During the annual dry season, *A*. *gayanus* biomass senesces and the litter remains as part of the standing tussock, unlike native grasses that have a higher proportion of litter in contact with the soil surface (Fig. 1)^29,30^. There are two main fates for *A*. *gayanus* biomass; being burnt or decomposed^31^. Whatever biomass is not burnt or decomposed is carried over to the next year, and can accumulate to up to 30 t ha^−1^ ^26,27^. We have previously documented that *A*. *gayanus* increases the magnitude of fire-mediated N fluxes^11,13^. N fluxes during a high intensity fire were ∼22 and 16.6 kg N ha^−1^ yr^−1^ in *A*. *gayanus* invaded and native grass savanna, respectively^13^. However, the fate of litter in years without fire has not been quantified. Understanding Ninputs to soil N pool via litter decomposition will advance understanding of the impact of *A*. *gayanus* invasion on the overall ecosystem soil N pool, particularly important considering this is an N-depauperate system^11,32^.

*A*. *gayanus* invasion increases the total amount of grass available for decomposition. Here we tested the hypothesis that *A*. *gayanus* invasion increases grass litter decomposition (mass loss) and therefore the total quantity of N released from the litter N pool, when compared to native savanna, and that this impacts on the soil N pool. We quantified *in situ* litter decomposition rates and litter N release using published data of harvested litter^11^. We conducted litterbag experiments, at soil surface and aerial positions, to examine the potential mechanisms driving differences between *in situ* litter decomposition of the standing litter, specifically differences in litter quality and habitat. We then used these rates to examine the impact of invasion on the soil total N pool over a 10-year period for three fire frequency scenarios.

## Results

### Production and in situ decomposition of grass litter

The *in situ* decomposition of the standing crop of grass litter was compared in native and invaded plots by repeat measurement in a 120-day period over the wet season and calculating the litter loss m^−2^. As reported in Rossiter-Rachor *et al*.^11^, the native and invaded plots had 41.5 ± 5.6 and 357.6 ± 43.9 g m^−2^ of litter respectively at the commencement of the wet season. By the end of the wet season, 61% of the *in situ* native grass litter had decomposed compared to only 30% in the invaded plots.

The daily litter mass loss (decomposition), and litter N loss (N release) was calculated for both native and invaded plots. Despite the lower proportion of *A*. *gayanus in situ* litter that decomposed, the larger quantity of litter meant that overall there was a significantly higher daily litter mass loss in invaded plots compared to native plots (mean 0.90 ± 0.41 *versus* 0.21 ± 0.04 g^−1^ m^−2^ day^−1^, respectively; Supplementary Table S1). The daily litter N loss (N release) was also significantly higher in invaded plots compared to native plots (mean 3.29 ± 1.49 *versus* 0.39 ± 0.23 mg g^−1^ N m^−2^ day^−1^), respectively; See Supplementary Table S1 for ANOVAs). The mechanisms explaining these differences in *in situ* litter decomposition are explained by differences in litter position (surface *versus* aerial, see below).

### Surface litter decomposition

**Figure 2:**
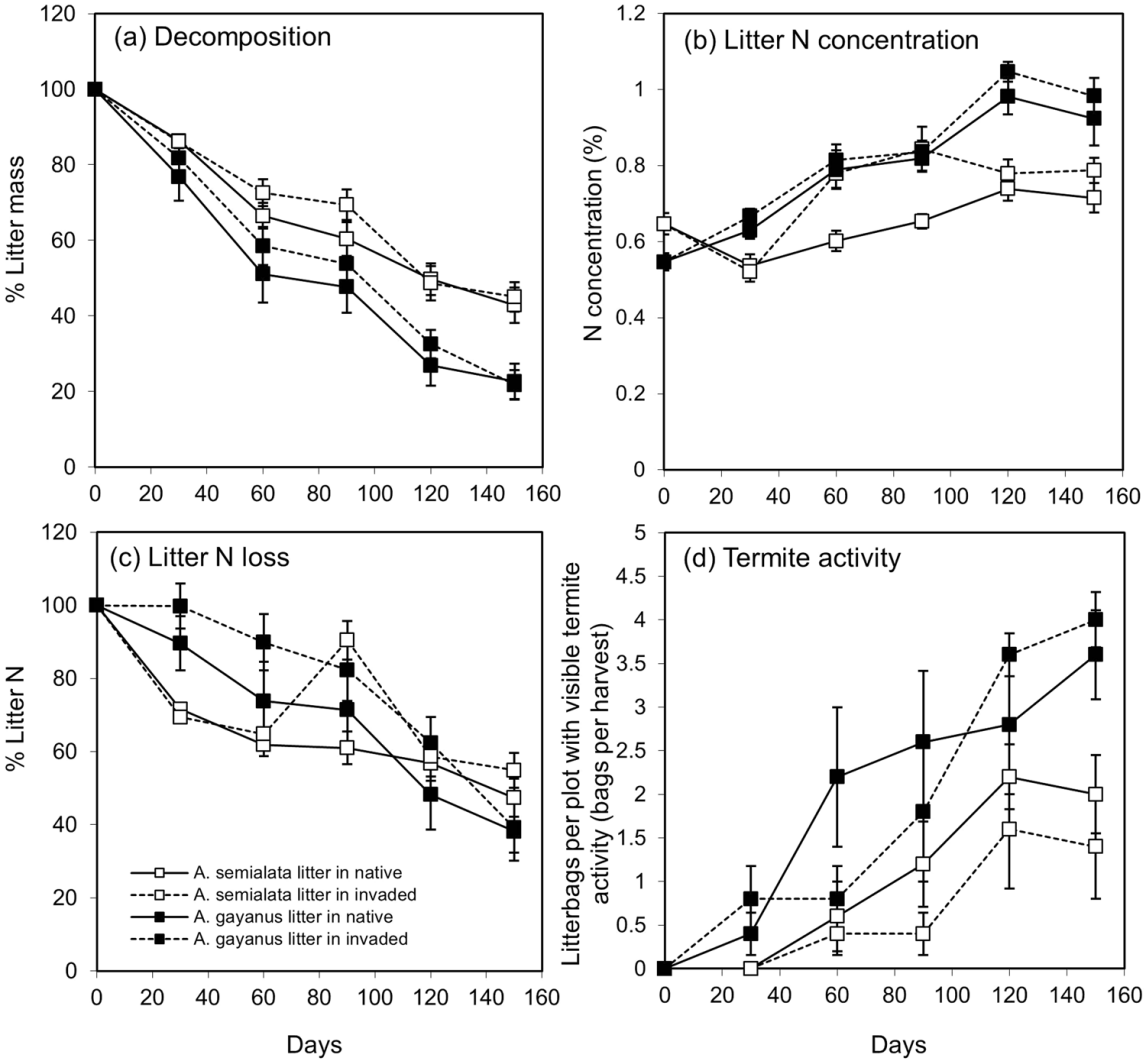
Surface litter (**a**) litter decomposition (% of initial mass remaining); (**b**) litter N concentration; (**c**) litter N release (% of initial N remaining) and (**d**) termite activity (bags per plot, per harvest), for *A*. *semialata* and *A*. *gayanus* litter at the soil surface, in native and invaded plots. Open symbols represent *A*. *semialata* litter and solid symbols represent *A*. *gayanus* litter. Solid lines represent native plots, and broken lines represent invaded plots. Values are means ± SE (*n* = 5).

A surface litterbag experiment was used, whereby litter from the native grass *A*. *semialata* and the invasive grass *A*. *gayanus* was incubated at the soil surface in: (1) their habitat of origin (native or invaded), and (2) their reciprocal habitats. At the beginning of the experiment the litter N and lignin concentrations of *A*. *gayanus* were significantly lower than those of the dominant native grass *A*. *semialata* (Table 1; Initial N *F*_[1,18]_ = 19.32, *P* = <0.001; Initial Lignin *F*_[1,18]_ = 15.33, *P* = 0.001), and had a significantly higher C:N ratio (Table 1; *F*_[1,18]_ = 17.35, *P* = 0.001). Litter mass declined rapidly for the first 60 days (from December to February) (Fig. 2a). After 150 days in the field, 21.8 ± 3.8 and 22.6 ± 4.8% of the initial *A*. *gayanus* litter remained in the invaded and native habitats, respectively (Fig. 2a). By comparison, 42.8 ± 4.7 and 45.1 ± 3.1% of initial *A*. *semialata* litter remained in the invaded and native habitats, respectively (Fig. 2a). The decomposition rate constant (*k*_*s*_) for *A*. *gayanus* litter was approximately double that of *A*. *semialata* (Table 2; *F*_[1,16]_ = 13.21, *P* = <0.01) with no significant effect of grass habitat type on *k*_*s*_.

**Table 1:** Initial litter characteristics of *A*. *semialata* and *A*. *gayanus* litter.

Litter type	*A. semialata*	*A. gayanus*
Initial N (%)	0.65 ± 0.28^a^	0.55 ± 0.22^b^
Initial C (%)	46.05 ± 0.09^a^	46.09 ± 0.09^a^
Initial C:N	72.91 ± 2.84^a^	86.19 ± 3.35^b^
Initial Lignin (%)	12.1 ± 0.4^a^	10.0 ± 0.4^b^
Initial Lignin:N	20.4 ± 0.6^a^	20.1 ± 1.0^a^

All values are means ± SE; *n* = 3 litter samples; *n* = 5 plot-pairs. Subscripts denote significantly different means.

**Table 2:** Decomposition rate constants (*k*_*s*_, yr^−1^) of *A*. *semialata* and *A*. *gayanus* litter in the surficial position in both native and invaded plots.

Litter type and grass habitat	*k*_*s*_ (yr^−1^)
*A*. *semialata* in native plots	2.12^a^
*A*. *semialata* in invaded plots	1.90^a^
*A*. *gayanus* in native plots	3.59^b^
*A*. *gayanus* in invaded plots	3.13^b^

Subscripts denote significantly different means.

The N concentration of the surface litter increased over the duration of the study in both *A*. *gayanus* and *A*. *semialata* (Fig. 2b). While both species displayed the same general trend, the N concentration of *A*. *gayanus* litter was significantly higher than that of *A*. *semialata* litter by the end of the study, indicating a greater immobilisation of N (Fig. 2b and Supplementary Table S2). The magnitude of difference in litter N concentration between litter types was higher at some plot-pairs, resulting in a significant plot-pair × litter type interaction (Supplementary Table S2). Litter N loss (expressed as the percentage of initial litter N pool remaining) was significantly greater in *A*. *gayanus* compared to *A*. *semialata* (Fig. 2c and Table S2). By the end of the reciprocal litterbag transplant experiment (May, beginning of dry season) the mean litter N pool for *A*. *gayanus* had decreased by 38.2 ± 9.6 and 39.1 ± 7.1% (invaded and native habitats, respectively) and 47.4 ± 4.8 and 54.9 ± 5.5% of the initial N pool for *A*. *semialata* litter (in invaded and native habitats respectively, Fig. 2c). There was no significant effect of grass habitat type on litter N loss, but there was a significant interaction between habitat and litter type at some sampling times (Supplementary Table S1).

Termite activity was more commonly associated with *A*. *gayanus* litter (Fig. 2d). By the end of the study 72 ± 10 and 80 ± 6% of *A*. *gayanus* litterbags had visible termite activity (in native and invaded habitats), compared to only 28 ± 12 and 40 ± 9% of *A*. *semialata* litterbags (in native and invaded habitats) (Fig. 2d). The mass loss of litter was highly correlated with termite activity in both litter type and habitat combinations (*A*. *semialata* litter in native grass habitat R^2^ = 0.91; *A*. *semialata* litter in *A*. *gayanus* habitat R^2^ = 0.94; *A*. *gayanus* litter in native grass habitat R^2^ = 0.90; *A*. *gayanus* litter in *A*. *gayanus* habitat R^2^ = 0.87). Only one termite species, *Nasutitermes eucalypti* (Mjoberg), was found feeding on litter in the litterbags.

### Aerial litter decomposition

**Figure 3:**
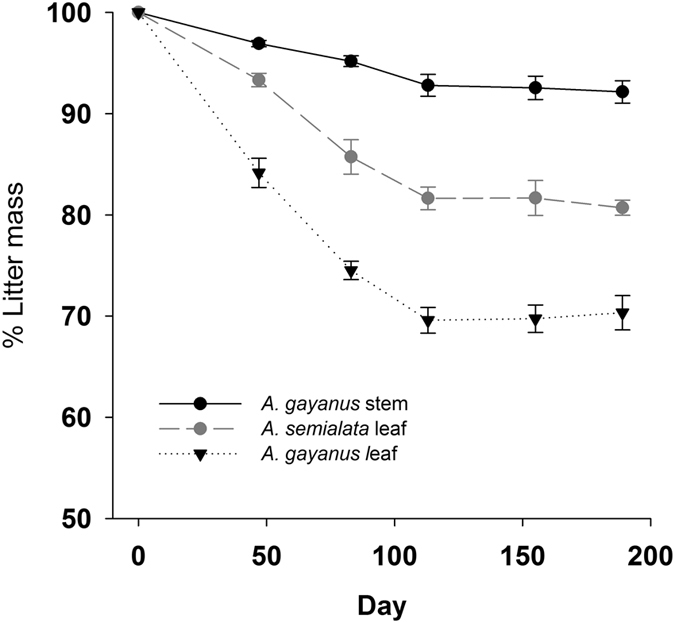
Aerial decomposition (% of initial mass remaining) of *A*. *semialata* and *A*. *gayanus* leaf litter, and *A*. *gayanus* stems at aerial positions. (Note: *A*. *semialata* stems were unable to be sampled due to their small size). Open symbols represent *A*. *semialata* litter and solid symbols represent *A*. *gayanus* litter. Values are means ± SE (*n* = 3).

An aerial litterbag experiment was used, whereby litter from *A*. *semialata* and *A*. *gayanus* was incubated at 1 m high in their habitat of origin (native or invaded). The results of the aerial litterbag decomposition experiment showed that litter mass declined more slowly in the aerial position and after 189 days in the field (November to May), with 80.7% of original *A*. *semialata* leaf, 70.3% of the *A*. *gayanus* leaf and 92.2% *A*. *gayanus* stem remaining by the end of the study period (Fig. 3). The magnitude of difference in litter mass loss between litter types was higher at some plot-pairs, resulting in a significant plot-pair × litter type interaction (Supplementary Table S3). The decomposition rate constant for aerial incubations (*k*_*a*_) was significantly greater for *A*. *gayanus* leaf, compared to *A*. *semialata* leaf, while decomposition of *A*. *gayanus* stems was significantly lower than the leaf of either species (*F*_[2,6]_ = 83.3, *P* = <0.001, Table 3).

**Table 3:** Aerial decomposition rate constants (*k*_*a*_, yr^−1^) of *A*. *semialata* and *A*. *gayanus* leaf litter, and *A*. *gayanus* stems.

Litter type	*k*_*a*_ (yr^−1^)
*A*. *semialata* leaf	0.504^a^
*A*. *gayanus* leaf	0.913^b^
*A*. *gayanus* stem	0.186^c^

Subscripts denote significantly different means.

### Implications for N cycling

**Figure 4:**
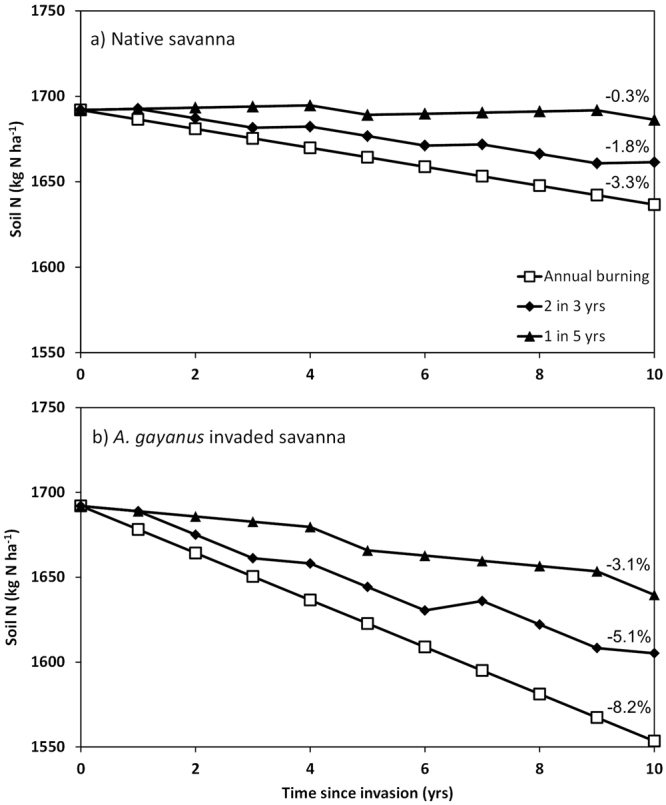
Predicted soil total N pool (Kg N ha^−1^) in (**a**) native savanna and (**b**) *A*. *gayanus* invaded savanna over a 10 year period, as a function of fire regime. Net soil N pool calculated by summing N inputs (fixation, wet deposition, N release from decomposition) and N outputs (fire losses, grass N uptake) in burnt and unburnt years for each fire regime. Three fire regimes were used; annual burning; fire 2 in 3 years (typical fire regime) and 1 in 5 years. Numbers indicate %N loss of gain relative to the initial total soil N pool. Soil N, grass N uptake under burnt and unburnt conditions, and fire N loss data were taken from Rossiter-Rachor *et al*.^11,13^. See Table 4 for further information on N inputs and N outputs in burnt/unburnt years.

In Table 4 we present the dominant inputs and output fluxes to the soil N pool to predict decadal consequences of invasion on N cycling. This table consists of fluxes quantified during this and previous studies at this location. We have compiled soil N fluxes for both a burnt and an unburnt state, and for both grass types. We include N input via rainfall, which for this region, is low at approximately 2 kg N ha^−1^ ^33^ and N input via fixation, which is largely unknown in these savannas, but is likely to be small^10^. The other significant soil N input is from grass decomposition and subsequent N release as quantified by this study (Table 4). Outputs from the soil N pool include uptake via plant growth and incorporation into biomass, and for the burnt condition, loss of N via biomass combustion and volatilisation and ash loss^13^. These annual input and output fluxes were summed over a 10 year period to examine the impact on soil N pool as a function of grass type and three fire regimes: (1) annually burnt (2) burnt 2 in 3 years, and (3) 1 in 5 years. The estimated annual N pool is plotted in Fig. 4 for each grass type and fire regime.

**Table 4:** Annual N fluxes (kg N ha^−1^ year^−1^) to the soil N pool in burnt and unburnt native and invaded savanna at Mary River National Park, Northern Territory Australia.

Fluxes (Kg N ha^−1^ year^−1^)	Native	Invaded	Source
**(A) UNBURNT YEAR**			
**Inputs**			
Wet deposition (via Rainfall)	2	2	Noller *et al*.^33^
Grass litter decomposition (*In situ*)	0.4	3.6	This study
**Total**	**2.4**	**5.6**	
**Outputs**			
N uptake (via plant growth)	1.7	8.6	Rossiter-Rachor^30^
N losses via fire (from grass layer)	0	0	(*No fire losses*, *as unburnt year*)
**Total**	**1.7**	**8.6**	
**(B) BURNT YEAR**			
**Inputs**			
Wet deposition (via Rainfall)	2	2	Noller *et al*.^33^
Grass litter decomposition (*In situ*)	0.6	1.4	Decomposition estimated from this study*
**Total**	**3.3**	**4.1**	
**Outputs**			
N uptake (via plant growth)	3.5	6.4	Rossiter-Rachor^30^
N losses via fire (from grass layer)	5.4	11.6	Rossiter-Rachor *et al*.^13^
**Total**	**8.9**	**18.0**	

These data were used to estimate the net effect on the soil N pool (kg N ha^−1^) integrating the fluxes over a 10 year period, for the three contrasting fire regimes: (1) annually burnt (2) burnt 2 in 3 years, and (3) 1 in 5 years (see Fig. 4).

*Decomposition estimated from this study (from ratio of production to *in situ* decomposition).

At the native savanna plots, the mean total soil N pool was 1692 ± 10 kg N ha^−1^ (0–30 cm^13^) a value we assume represents the native savanna soil N pool. Satellite monitoring of fires occurrence indicate that this location experiences a regime of one fire in five years (NAFI, http://www.firenorth.org.au, accessed 8 December, 2016). Using this fire regime and soil N inputs (rainfall, litter N release estimates) and outputs (plant N uptake, N loss from fire, Table 4), the total soil N pool in native savanna would be largely stable after a decade (0.3% loss) while invaded savannas would experience a 3.1% loss in soil N (Fig. 4). This decline occurs despite N inputs over 10 years from rainfall and 4.6 kg N ha^−1^ and 31.4 kg N ha^−1^ due to litter decomposition in the native and invaded savanna respectively. However, as fire frequency increases, the soil N input via litter decomposition declines and loss via fire increases. If the savanna was burnt annually, or burnt two in every three years (a fire regime more typical of high rainfall Australian savannas^31^, the loss from the N pool is more substantial in invaded areas compared to native areas (Fig. 4). This is because N loss through volatilisation is twice as great following invasion (Table 4). An annual fire regime in invaded areas results in an 8.2% loss in the total soil N pool after a decade (Fig. 4).

## Discussion

We confirm that *Andropogon gayanus* invasion results in marked changes to litter decomposition and concomitant N fluxes. Compared to native grasses, *A*. *gayanus* produced more litter. However, the majority (>80%) of *A*. *gayanus* litter was distributed in the aerial position (between 30 cm and 4 m off the ground) whereas 100% of native grass litter was distributed within 30 cm of the soil surface (Fig. 1). Only 30% of the *A*. *gayanus in situ* litter decomposed over the wet season compared to 61% of the native grass litter, resulting in a considerable amount of aerial *A*. *gayanus* litter being carried over into the dry (and fire) season. Standing aerial litter decomposes slower than litter close to the ground due to lower moisture availability and reduced presence of decomposers communities. *A*. *gayanus* stems were particularly resistant to decay, with only ∼8% of stem material decomposing over the wet season. The accumulation of standing litter in African C_4_ pasture grass swards is well documented in South American pasture systems^34^. Similarly, in Australian savannas, this resistance to aerial litter decomposition results in the accumulation of large quantities of litter in invaded plots, with up to 30 t ha^−1^ of grass litter in long unburnt *A*. *gayanus* invaded systems^26^, compared to up to 5.8 t ha^−1^ in long unburnt native grass savanna^10^.

Litter quality also impacted on decomposition rates. In tropical wet seasons, litter quality, not climate, is the key determinant of litter decomposition rates, as temperature and moisture are generally non-limiting in the wet season^9,35–37^. Litter quality parameters include initial litter N concentrations, lignin concentrations, C:N and lignin:N ratios^19^. Soil surface decomposition rates (*k*_*s*_) of *A*. *gayanus* were approximately double that of *A*. *semialata* litter. *A*. *gayanus* litter had significantly lower N and lower lignin concentration than native grass litter (Table 1). While live *A*. *gayanus* leaf tissue has a higher N concentration than native grasses (Rossiter-Rachor *et al*.^11^), it is highly effective at translocating this N from senescing tissues, leaving *A*. *gayanus* litter that has a low N, and a low lignin concentration. Lignin concentration has been found to be negatively correlated with the rates of litter decomposition in several tropical grasses, including *A*. *gayanus* ^37^. Our lignin results for *A*. *gayanus* (10 ± 0.4%) were within the range reported by Thomas & Asakawa^37^ for *A*. *gayanus* (9.2–11.7%). The relatively low litter quality of *A*. *gayanus* reported in our study is common among C_4_ grasses^38^, and may result in litter in the early stages of decomposition taking up and storing N^39–41^. Bacteria and/or fungi take up N to supplement the N in the litter being decomposed^42^. N immobilisation is particularly common in C_4_ grasses^21,37^. Importantly, our results highlight that changes in litter decomposition can occur as soon as the invasion commences, as the altered litter decomposition rates were driven by the altered litter quality of *A*. *gayanus*, not the habitat in which decomposition occurred.

Litter quality is also likely to have influenced termite feeding preferences, which preferentially consumed *A*. *gayanus* litter, regardless of the habitat the litterbags were incubated in (native or invaded savanna), a finding that is consistent with studies of *A*. *gayanus* in its native range in Nigeria^43,44^. In Africa, the termite *Trinervitermes geminatus* (Wasmann) had a stronger preference for *A*. *gayanus* litter than five other African grasses tested, even though the density of *A*. *gayanus* stands at their study sites was significantly lower than the other grasses. It remains unknown which properties of *A*. *gayanus* litter are responsible for such preferential behaviour by termites.

The high decomposition rates found in this study are comparable to those reported in the literature for C_4_ African grasses; with *k* values ranging between 0.0020 g g^−1^ d^−1^ for *Brachiaria dictyoneura* ^37^, 0.0025 g g^−1^ d^−1^ for *A*. *gayanus* ^37^ and 0.0174 g g^−1^ d^−1^ for *Panicum maximum* ^45^. The *A*. *gayanus k*_*s*_ values from this study were (0.0089–0.0113 g g^−1^ d^−1^) were at the higher end of this range.

### Implications for soil N fluxes

This study demonstrates that replacing native savanna grasses with *A*. *gayanus*, and altering the ecosystem process of litter decomposition, has important implications for soil N cycling. Overall, the N flux data shows that the increased litter N release from *A*. *gayanus* (*in situ* decomposition data) does not compensate for the increased outputs from the soil N due to fire mediated N losses^13^ or increased N uptake by *A*. *gayanus* ^11^ given the ambient fire regime. Longer-term (decadal) implications of changed N relations are a reduction in soil total N which will increase with fire frequency. In high rainfall Australian savannas impacted by invasive grasses, fire frequency is typically annual or biennial burning^2^. In the absence of additional inputs via N fixation, this fire regime would reduce total soil N by up to 8.2% in invaded savanna within a decade (Fig. 4). This is likely to have substantial implications given that the savannas studied here are among the most N-depauperate systems globally (Table 4). However, to date, we have not detected a consistent change in soil N over time following invasion^30^. This may be due to the death and decomposition of woody vegetation in the invaded areas^24^ that is co-occurring as a consequence of invasion and increased fire intensity, and this process would be releasing substantial amounts of N. The N losses may be compensated for by N fixation, with speculation^46^ but no detailed investigation that *A*. *gayanus* encourages diazotrophic soil microbes. Clearly, longer-term studies of extremely impacted *A*. *gayanus* sites (i.e. where fires have significantly reduced the presence of live trees^25^) are needed to quantify the consequences of *A*. *gayanus* invasion on whole of ecosystem N balance in invaded savanna ecosystems.

## Methods

### Site description and experimental design

Our research was conducted in Mary River National Park (Northern Territory, Australia (12°64′S, 131°75′E). Temperatures are high throughout the year (27 °C, annual average), while rainfall is highly seasonal (1591 mm, annual average, Weather station number 014263, Australian Bureau of Meteorology) and concentrated in the wet season between October and April. The savanna overstorey is dominated by *Eucalyptus miniata* (Cunn. Ex Schauer) and *Eucalyptus tetrodonta* (F. Muell), with an understory dominated by native perennial grasses *Alloteropsis semialata* (R. Br.) Hitchc. and *Eriachne triseta* Nees ex Steud., with patches of annual grasses, including *Pseudopogonatherum irritans* (Br.) A. Camus. *Andropogon gayanus* (gamba grass) has invaded extensive areas of the park, replacing the short savanna grass communities (~0.5 m) with dense, tall, almost monospecific swards up to 4 m high^47^.

We compared litter decomposition of *A*. *semialata* and *A*. *gayanus* using a randomised block design. Paired-plots (blocks) were established, with each plot-pair consisting of an area dominated by native grass (hereafter referred to as ‘native’ plots), and a nearby (approximately 50 m distance) *A*. *gayanus* dominated area (hereafter referred to as ‘invaded’ plots). Plot-pairs were located up to 600 m apart, and each plot was 50 m × 50 m in size. In native plots, the grass component included several native grass species, while *A*. *gayanus* was the only grass species present in invaded plots.

### Litter decomposition

We used two complementary methods based on the loss rate of grass litter over time to quantify litter decomposition. We followed an *in situ* cohort of native grass and *A*. *gayanus* litter through a wet season to quantity the decomposition rate based on litter loss over time. In addition, we used litterbags located on the soil surface and also suspended aerially at 1 m in height to quantify decomposition rate constants (*k*) for litter exposed to microbial, fungal and invertebrate decomposition (surface) *versus* exposure to microbial, fungal and photodegradation (aerial). The wet season is the peak period of decomposition in these savannas due to the high rainfall and humidity^48^ and very little new grass litter is produced during this time.

#### Quantification of in situ litter decomposition and litter N loss

The *in situ* decomposition of the standing crop of grass litter was compared in native grass and invaded plots by following the cohort of grass litter over a wet season. Plots were not burnt during the study period and any reduction in litter mass between sampling events was assumed to be due to decomposition of the previous year’s grass production^11,19^, because very little new grass litter is produced during the wet season^11^. We used litter data published in Rossiter-Rachor *et al*.^11^ to calculate the litter loss over time. As described in Rossiter-Rachor *et al*.^11^, grass phytomass (biomass + necromass) was destructively harvested at the beginning (November 2002) and then end (March 2003) of the wet season, some 120 days later. Samples were collected from three random replicate 2 × 2 m quadrats, within each of the five plot pairs (n = 15 quadrats per grass type, per sampling time). Samples were returned to the lab, and sorted into green leaves and stems (live biomass, data not presented) and dead standing grass (necromass; hereafter referred to as grass litter). All litter was dried for 48 h at 60 °C and weighed. Subsamples were ground in a Wiley mill, analysed for %N and %C using a Carlo Erba analyser (Thermo Electron, MA, USA), and the initial litter N pool calculated. In this study we calculated the *in situ* grass litter decomposition as the decrease in grass litter mass between November 2002 and March 2003. Similarly, litter N release (product of litter mass x litter tissue N concentration; g N m^−2^; following)^49^ was calculated as the change in the grass litter N pool during this period. This litter N release was also expressed as a release rate per day (calculated as the decrease in the litter N pool, divided by the number of days between the November and March harvests).

#### Quantification of surface litter decomposition rate constant

We quantified surface litter decomposition using a reciprocal transplant experiment following Rothstein *et al*.^50^ in which leaf litterbags were incubated in the field in plot pairs. Senesced A. semialata and A. gayanus leaf litter was collected from the site in November 2003, dried at 60 °C to a constant mass and 5 g sub-samples were sewn into 15 × 15 cm mesh bags (∼1.5 mm mesh size). Fifteen sub-samples were ground in a Wiley mill, analysed for %N and %C using a Carlo Erba analyser (Thermo Electron, MA, USA), and the initial litter N pool calculated. Samples were also analysed for % Acid Detergent Lignin using an Ankom A200 fibre analyser (Ankom Technology, NY, USA). Litterbags were collected every thirty days up to 150 days (7^th^ January, 6^th^ February, 7^th^ March, 6^th^ April and 6^th^ of May 2004, respectively). A total of 1509.1 mm rainfall was recorded during this period^51^. At each harvest date, 50 litterbags of both grasses were retrieved (5 plot-pairs × 2 grass habitats × 5 replicates). Litter was carefully washed (without pressure) with de-ionised water to remove soil, roots or live material (following)^50^, before being dried at 60 °C and weighed for mass loss. Litter decomposition was expressed as percent of the initial mass remaining. We used time series of litter mass and N concentrations to calculate k_s_ for A. gayanus and A. semialata surface litter using a simple negative exponential decay model^52^;

1 $${M}_{{\rm{t}}}/{M}_{{\rm{i}}}=m\,.\,{{\rm{e}}}^{{\textstyle \text{-}}kt}$$

where *M*_*t*_ is the litter mass at time *t* (years), *M*_i_ the initial litter mass, *k* the decomposition rate constant for surface litter and *m* is a regression constant.

We randomly chose three bags of each litter type, from each plot-pair, at each sampling time. Litter was ground, analysed for %N and the litter N pool (litter mass × N concentration) was calculated for each harvest date. Litter N release was expressed as percent of the initial litter N pool remaining, showing net losses (mineralisation) or gains (immobilisation). During collection of the litterbags, visible termite activity was noted via evidence of termites in the litterbags, soil sheeting attached to the litterbags and damage to the side of the litterbag in contact with the soil. Termites present in the litterbags were collected, placed in 95% ethanol, and sent to the CSIRO, Darwin laboratories for identification.

#### Quantification of aerial litter decomposition rate constant

We quantified aerial decomposition rates of leaf and stem litter using an aerial litterbag experiment following Liao *et al*.^19^. *A*. *semialata* and *A*. *gayanus* leaf litter, as well as *A*. *gayanus* stem litter, was collected in November 2014, and processed into litterbags as previously described. We placed litterbags in 3 plot-pairs in early December 2014, to coincide with the first wet-season rains. Nine litterbags of *A*. *semialata* leaf litter were placed in each native plot. In invaded plots, nine *A*. *gayanus* litterbags of leaf litter, and nine of *A*. *gayanus* stem were incubated. We attached litterbags to aluminium stakes 1 m in height. Three randomly selected bags were collected from each plot after 47, 83, 113, 155 and 189 days in the field respectively (3^rd^ February, 11^th^ March, 10^th^ April, 22^nd^ May, 25^th^ June). A total of 1282.2 mm of rain was recorded during this period^51^. Litterbags were processed as previously described and the decomposition rate constant for aerial litters (*k*_*a*_) was calculated as per Equation 1.

### Impacts of invasion and fire on soil N cycling

The effect of *A*. *gayanus* invasion and fire on the soil N pool was investigated by collating measured N fluxes for native grass and invaded savanna. The initial N pool was assumed to be 1692 kg ha^−1^ (0–30 cm) as measured by Rossiter-Rachor *et al*.^13^. The soil N inputs included wet deposition, litter N release from *in situ* decomposition, with N outputs including fire losses and grass N uptake from grass growth. These N inputs and outputs for burnt and unburnt years., and these data were used to investigate the net effect of different fire resgimes on the soil N pool. Three fire regimes used; annual burning; fire 2 in 3 years (as occurs at the study location) and 1 in 5 years. Changes to the soil N pool were estimated over a 10-year period by summing net N inputs and outputs as described above for burnt and unburnt years for each fire regime.

Data describing the initial soil N pool and grass N uptake rate under burnt and unburnt conditions, and fire loss data were calculated using the data presented in Rossiter-Rachor *et al*.^11,13^. Grass N uptake was estimated by summing annual increments of biomass N (see ref.^11^ for details of biomass N determination). N input via rainfall was based on data of Noller *et al*.^33^ in a study conducted in the nearby (~100 km east) Alligator River Region of the Northern Territory; a region very comparable in climate, vegetation and fire regime to that of the current study.

### Statistical analysis

*In situ* litter decomposition rates were compared using a two-factor analysis of variance (ANOVA), with factors litter type (*A*. *semialata* or *A*. *gayanus*, fixed) and plot-pair (5 locations, random). Differences in surface litter decomposition rate, litter N concentration, and litter N release were analysed using a four-way mixed model ANOVA, with factors time (5 levels, fixed), plot-pair (3 locations, random), grass habitat type (native or invaded, fixed), and litter type (*A*. *semialata* or *A*. *gayanus*, fixed). Differences in aerial litter decomposition rates were analysed using a three-way ANOVA, with the factors time (5 levels, fixed), plot-pair (5 locations, random), and litter type (*A*. *semialata* or *A*. *gayanus*, fixed). Before statistical analyses were undertaken, assumptions of ANOVA were checked using Cochran’s test, and where necessary data were transformed prior to analyses to improve normality and homogeneity. Tukey’s pairwise contrasts were used to explore the main effects. Analyses were carried out using Systat Version 10 (SPSS, Chicago, IL, USA).

## Electronic supplementary material

Supplementary information
