# Supplementary material for: Invasive Andropogon gayanus (Gamba grass) alters litter decomposition and nitrogen fluxes in an Australian tropical savanna
Source: Sci Rep. 2017 Sep 15;7:11705. doi: 10.1038/s41598-017-08893-z (PMC5601926; doi:10.1038/s41598-017-08893-z)
Supplement: Supplementary file 1 — Supplementary information [file 41598_2017_8893_MOESM1_ESM.doc]

Supplementary Information

# **Invasive *Andropogon gayanus* (Gamba grass) alters litter decomposition and nitrogen fluxes in an Australian tropical savanna**

N. A. Rossiter-Rachor^1^*, S. A. Setterfield^2^, L. B. Hutley^1^, McMaster, D^1^, Schmidt, S^3^ and M. M. Douglas^2^

^1^ Charles Darwin University, Darwin, Northern Territory, 0909, Australia

^2^ University of Western Australia, Crawley, Western Australia 6099, Australia

^3^ The University of Queensland, Brisbane, Queensland 4072, Australia

*Corresponding author; Natalie Rossiter-Rachor

Email: [natalie.rossiter@cdu.edu.au](mailto:natalie.rossiter@cdu.edu.au)

Tel: +61-8-8946-6469

Table S1: Summary of significant results from a two-factor ANOVA on wet season *In situ* litter (a) Daily litter mass loss (g m^2^ day^-1^) and (b) Daily litter N loss (mg N m^2^ day^-1^); of native grass and *A. gayanus* litter. “Litter type” refers to whether the grass litter was native grass or *A. gayanus*, “Plot-pair” refers to the location. Only significant results are indicated.

| Source | d.f | MS Effect | *F* | *P* |
| --- | --- | --- | --- | --- |
|  |  |  |  |  |
| **(a) Daily litter mass loss** |  |  |  |  |
| Litter type | 1 | 3.6 | 4.8 | 0.040 |
| Plot-pair | 4 | 2.5 | 3.3 | 0.031 |
| Litter type x Plot-pair | 4 | 2.8 | 3.8 | 0.019 |
|  |  |  |  |  |
| **(b) Daily Litter N loss** |  |  |  |  |
| Litter type | 1 | 38.0 | 15.2 | 0.002 |
| Plot-pair | 2 | 25.8 | 10.3 | 0.002 |
| Litter type x Plot-pair | 2 | 41.0 | 16.4 | <0.001 |

Table S2

Summary of significant results from a four-factor ANOVA on surface litter (a) Litter decomposition (% initial litter mass remaining) (b) Litter N concentration (% N), and (c) Litter N loss (% of initial litter N pool remaining); of *A. semialata* and *A. gayanus* litter, in native and invaded plots. “Time” refers to the harvest date, “Plot-pair” refers to the location, “grass habitat” refers to whether the plot was native grass or invaded, “Litter type” refers to whether the grass litter was *A. semialata* or *A. gayanus*. Only significant results are indicated.

| Source | d.f | MS Effect | *F* | *P* |
| --- | --- | --- | --- | --- |
| **(a) Litter mass loss** |  |  |  |  |
| Time | 4 | 38801.9 | 262.6 | <0.0001 |
| Plot-pair | 4 | 6499.7 | 13.1 | <0.0001 |
| Litter type | 1 | 29455.1 | 8.5 | 0.043 |
| Plot-pair x Litter type | 4 | 3454.7 | 6.9 | <0.0001 |
| Plot-pair x Litter type x Habitat type | 4 | 1652.1 | 3.3 | 0.010 |
|  |  |  |  |  |
| **(b) Litter N concentration** |  |  |  |  |
| Time | 4 | 0.512 | 42.7 | <0.0001 |
| Plot-pair | 2 | 0.036 | 3.6 | 0.030 |
| Litter type | 1 | 1.383 | 29.4 | 0.032 |
| Plot-pair x Litter type | 2 | 0.036 | 3.7 | 0.028 |
| Plot-pair x Habitat type | 2 | 0.047 | 4.8 | 0.010 |
| Plot-pair x Litter type x Habitat type | 8 | 0.031 | 3.2 | 0.003 |
|  |  |  |  |  |
| **(c) Litter N loss** |  |  |  |  |
| Time | 4 | 10078.1 | 19.8 | <0.0001 |
| Plot-pair | 2 | 4127.2 | 4.6 | 0.012 |
| Litter type | 1 | 41585.7 | 2519.7 | <0.0001 |
| Time x Litter type | 4 | 2039.8 | 4.4 | 0.035 |
| Time x Habitat type x Litter type | 4 | 1680.4 | 6.8 | 0.011 |

**Table S3** Summary of significant results from a three-factor ANOVA on aerial litter decomposition (% initial litter mass remaining) of *A. semialata* leaf, *A. gayanus* litter, and *A. gayanus* stem. “Time” refers to the harvest date, “Plot-pair” refers to the location, “Litter type” refers to whether the litter was *A. semialata* leaf, *A. gayanus* leaf or *A. gayanus* stem. Only significant results are indicated.

| Source | d.f | MS Effect | *F* | *P* |
| --- | --- | --- | --- | --- |
| **(a) Litter mass loss** |  |  |  |  |
| Time | 4 | 539.2 | 46.2 | <0.001 |
| Litter type | 2 | 4603.9 | 394.5 | <0.001 |
| Litter type × Plot-pair | 4 | 48.7 | 4.2 | 0.004 |
